# Carotid Resistance and Pulsatility: Non-Invasive Markers for Diabetes Mellitus-Related Vascular Diseases

**DOI:** 10.3390/jcm14072523

**Published:** 2025-04-07

**Authors:** Chun-Chieh Liu, Chao-Liang Chou, Chuen-Fei Chen, Chun-Fang Cheng, Shu-Xin Lu, Yih-Jer Wu, Tzu-Wei Wu, Li-Yu Wang

**Affiliations:** 1 Cardiovascular Center, Department of Internal Medicine, MacKay Memorial Hospital, Taipei City 104, Taiwan; jasonliu0528@gmail.com (C.-C.L.);; 2 Department of Medicine, MacKay Medical College, New Taipei City 252, Taiwan; chaoliangchou@gmail.com (C.-L.C.); cfchen@mmc.edu.tw (C.-F.C.); 3 Department of Neurology, MacKay Memorial Hospital, Taipei City 104, Taiwan; 4 Department of Health, New Taipei City Government, New Taipei City 251, Taiwan; aa3310@ntpc.gov.tw

**Keywords:** extracranial carotid artery, pulsatility index, resistance index, case–control study, community-based, diabetes mellitus, association study

## Abstract

**Background:** Diabetes mellitus (DM) is a major determinant of aging-related vascular diseases. The arterial pulsatility index (PI) and resistance index (RI) are biomarkers of vascular aging. The available data regarding DM with arterial PI and RI are limited. The specific aim of this study was to explore the relationships between DM and the segment-specific PI and RI of the extracranial carotid arteries. **Methods:** We enrolled 402 DM cases and 3416 non-DM controls from a community-based cohort. Each subject’s blood flow velocities in the extracranial common (CCA), internal (ICA), and external (ECA) carotid arteries were measured by color Doppler ultrasonography and used to calculate PIs and RIs. **Results:** The DM cases had significantly higher age–sex-adjusted means of carotid RIs and PIs than the non-DM controls (all *p*-values < 0.005). After controlling for the effects of conventional cardio-metabolic risk factors, all carotid RIs and PIs remained significantly correlated with higher odds ratios (ORs) of having DM. The relationships with DM were stronger and more significant for the ECA RI and PI. The multivariable-adjusted ORs were 1.36 (95% confidence interval [CI], 1.21~1.54, *p* = 3.9 × 10^−7^) and 1.30 (95% CI, 1.17~1.45, *p* = 8.7 × 10^−7^) for 1.0 SD increases in the ECA RI and PI, respectively. Compared to the best fit model of conventional cardio-metabolic risk factors, the additions of the ECA RI and PI significantly increased the area under the receiver operating characteristic curve by 0.85% (95% CI, 0.11~1.59%; *p* = 0.023) and 0.69% (95% CI, 0.01~1.37%; *p* = 0.046), respectively. **Conclusions:** This study shows significantly positive associations between DM and carotid RIs and PIs. Carotid RIs and PIs are potential biomarkers for DM-related vascular diseases.

## 1. Introduction

Diabetes mellitus (DM) is a current global health concern. In 2021, the estimated number of global DM cases was 536.6 million among adults aged 20–79 years [1]. More than one tenth of adults aged 20–79 years were affected with DM in 2021. The global age-standardized prevalence rate of DM was estimated to be 10.5% in 2021 [1]. Furthermore, DM is a major contributor to health expenditure and disease burden. The estimated global cost of DM in adults aged 20–79 years was USD 1.32 trillion in 2015 and will increase to USD 2.48 trillion in 2030 [2]. The estimated number of global disability-adjusted life-years (DALYs) attributed to DM rose from 52.7 million DALYs in 2010 to 78.9 million DALYs in 2021 [3]. The global population will be aging rapidly during the next two decades. Additional, DM is one the major determinants of several vascular diseases, including stroke and cardiovascular disease (CVD) [4,5,6,7]. Consequently, increases in DM-related impacts in the near future seem to be inevitable [1]. Early detection and prompt diagnosis of DM are among the key elements of reducing the rapid increase in DM-related impacts [8]. In addition to measuring blood glucose levels by standardized methods, early detection of DM may be aided by diagnostic modalities.

Type II DM, the major type of DM that accounts for more than 90% of DM cases, is a complex disease. Etiologic studies have correlated the incidence of DM with multiple modifiable risk factors. There are consistent associations between a higher incidence of DM and low physical activity, sedentary behavior, and cigarette smoking [8]. However, a higher incidence of DM is also correlated with older age, female sex, and genetic factors [9], which are basically non-modifiable. DM is consistently and strongly associated with the incidences of several vascular diseases, including cardiovascular diseases [4], stroke [5,6], and peripheral arterial disease [7]. It is reasonable to hypothesize that there is a close link between the aging of the blood vessels and the incidence of DM.

Arterial stiffness occurs with the aging of the blood vessels [10,11]. Pulse wave velocity (PWV) can be assessed non-invasively and is considered the ‘gold standard’ measurement of arterial stiffness [12]. High PWV has been correlated with significantly higher risks of cardiovascular events and mortality as well as all-cause mortality [13,14]. However, the relationship between PWV and DM is inconsistent [15,16,17,18,19,20]. In addition, no reports have assessed the increased predictability caused by including PWV in the DM prediction model.

The arterial resistance index (RI) and pulsatility index (PI) are markers of arterial stiffness and can be assessed non-invasively by Doppler ultrasonography in the field [21]. Recent research has also correlated higher arterial PI or RI with a higher risk of cerebrovascular disease [22,23,24]. Additionally, several small clinical- or hospital-based studies have correlated RI and PI of the arteries, especially the middle cerebral artery (MCA), with DM [25,26,27,28]. To our knowledge, there are no data from large studies of the general population. Additionally, no study has compared the influence of RI and PI measurement at different segments of the extracranial carotid arteries, the most accessible large artery in the circulation system. In this large community-based study, we enrolled approximately 4000 adults aged 40–74 years and bilaterally assessed the RIs and PIs of their extracranial common (CCA), internal (ICA), and external (ECA) carotid arteries. The specific aim of the study was to explore the relationships between DM and segment-specific carotid RIs and PIs.

## 2. Materials and Methods

### 2.1. Study Subjects

**Figure 1:**
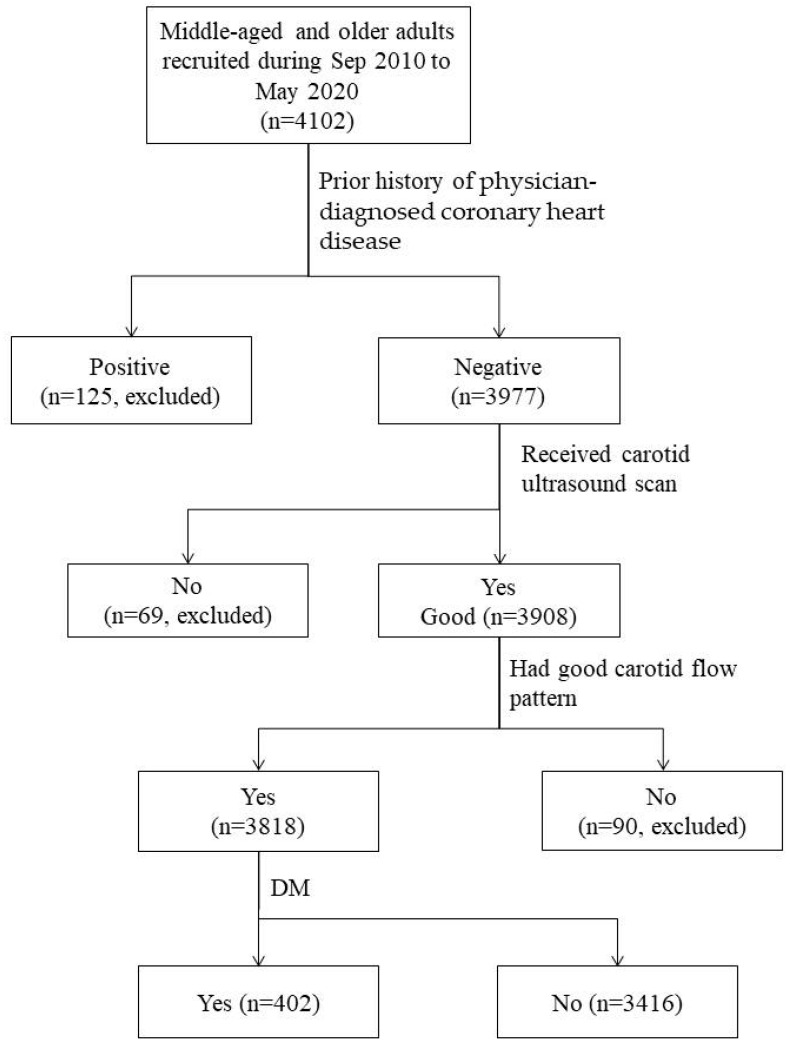
Steps of subject selection.

The study subjects were recruited from an ongoing community-based cohort study [29,30]. The eligibility for inclusion were those who aged 40–74 years and had resided in the studied communities for at least six months [29,30]. We used three measures to promote participation. The first was that we obtained the addresses of households with eligible subject(s) from the district offices of household registration. The second was that we sent highly informative invitation letters to eligible households. The invitation letters described the objective, protocols, inclusion and exclusion criteria, and main measurement methods of the study. The last was that, to increase accessibility, we set up recruitment sites in local public places. A total of 4102 eligible residents voluntarily provided informed consent and were enrolled from September 2010 to May 2020. Enrollees completed a structured questionnaire and provided a fasting blood sample for biochemical measurement. There were 125 enrollees who had a positive history of coronary heart disease (CHD) and were excluded. Additionally, 69 subjects who did not receive an ultrasonographic scan and 90 subjects who lacked a proper blood flow pattern were excluded. Consequently, in this community-based case–control study, there were 3818 middle-aged or older subjects, and 402 (10.5%) of them fitted the definition of DM (Figure 1).

The study complies with the 1975 Helsinki Declaration regarding ethics in medical research and was reviewed and approved by the institutional review boards of MacKay Medical College (No. P990001; date of approval: 5 July 2010) and MacKay Memorial Hospital (No. 14MMHIS075; date of approval: 23 May 2014).

### 2.2. Anthropometric and Biochemical Measurements

The measurements of baseline anthropometric and clinical characteristics have been described previously [29,30]. In brief, each subject’s systolic (SBP) and diastolic (DBP) blood pressure was measured three times, with an interval of 3 min or more, in the morning after 10 min of rest by using a digital system (UDEX-Twin; ELK Co., Daejon, Korea). For statistical analyses, the means of repeated measurements of SBP and DBP were used. Body mass index (BMI), an indicator of general obesity, was calculated as body weight (Kg) divided by square of body height (meter). Waist-to-hip ratio (WHR), an indicator of central obesity, was calculated as waist circumference (WC; cm) multiplied by 100% then divided by hip circumference (HC; cm).

For the biochemical measurements, a venous blood sample was drawn from each subject after ≥10 h of fasting. In this study, blood lipids and glucose were measured by an autoanalyzer (Toshiba TBA c16000; Toshiba Medical System, Holliston, MA, USA) with commercial kits (Denka Seiken, Tokyo, Japan). The biomarkers of blood lipids included fasting plasma triglycerides (FTG), total cholesterol (TCHO), and high-density (HDL-C) and low-density (LDL-C) lipoprotein cholesterol. The biomarkers of blood glucose include fasting plasma glucose (FPG) and HbA1c.

In this study, the definition of DM includes the following: (1) FPG ≥ 126 mg/dL, (2) HbA1c ≥ 6.5%, (3) diagnosis of DM by a physician, and (4) the use of insulin or other glucose-lowering medications. The definition of hypertension includes the following: (1) SBP ≥ 140 mmHg or DBP ≥ 90 mmHg, (2) diagnosis of hypertension by a physician, and (3) the use of antihypertensive medications. Subjects who had smoked cigarettes and drunk alcohol-containing beverages ≥4 days/week during the last month before enrollment were regarded as current cigarette smokers and current alcohol drinkers, respectively.

### 2.3. Ultrasonographic Measurements of Carotid Blood Flow

In the study, the blood flow velocities, including peak-systolic velocity (PSV), end-diastolic velocity (EDV), and time-weighted maximum flow velocity (MFV), of the extracranial carotid arteries were measured as described previously [31]. In brief, we scanned the bilateral extracranial carotid arteries of each subject using high-resolution color Doppler ultrasonography systems (GE Healthcare Logie E, Vivid 7, and Vivid E9; General Electric Company, Milwaukee, WI, USA) with the PW-Doppler mode. The ultrasonography systems were equipped with a linear array transducer L9-RS (3.33 to 10.0 MHz; General Electric Company, Milwaukee, WI, USA) and operated by two well-trained and experienced technicians who were unaware of each examinee’s clinical profile. Each participant was examined in the supine position with his/her head turned 45° from the site being measured. For all Doppler measurements, the insonation angle was ≤60° and the sample volume size covered 1/2 to 2/3 of the lumen of the middle segment of each carotid artery. In this study, subjects without 3 similar patterns of waveforms were regarded as lacking a proper flow pattern and excluded. Each subject’s PI and RI were automatically calculated using standard formulas:
PI=PSV-EDV, RI=PSV-EDV.MFVMFV

In the study, we used the averages of the right and left carotid PIs and RIs for statistical analyses.

### 2.4. Statistical Analyses

In this study, we used Student’s t-test to test the significance of differences in the means of continuous measurements between DM cases and non-DM controls. Logarithmic transformation was performed for continuous random variables with positive skewness. The significance of the associations between DM status and categorical variables was tested by the Chi-square test. The age–sex-adjusted mean differences in the carotid PIs and RIs between DM cases and non-DM controls were obtained by the generalized linear regression model. We used a three-stage procedure to assess the independent effects of carotid PIs and RIs on DM. First, we used logistic regression analyses with a stepwise selection method to obtain the best fit model of conventional CVD risk factors, designated as the base model. Second, each carotid PI and RI was separately added to the base model to assess its independent effect on DM. Lastly, all significant carotid PIs and RIs were included in the base model with a stepwise selection method to obtain the significantly independent indicator(s) of DM. In this study, the strengths of the associations between carotid PIs and RIs and DM were manifested by adjusted odds ratios (ORs). The area under the receiver operating characteristic curve (AUROC) was used as an indicator of the predictability of the DM prediction model. The difference in the AUROC between the base model and the models with carotid PIs and RIs was used as an indicator of increased predictability and its significance was tested by the Chi-square test. All statistical analyses were performed using SAS 9.4 (SAS Institute Inc., Cary, NC, USA).

## 3. Results

### 3.1. Baseline Anthropometric and Biochemical Measurements Between DM Cases and Non-DM Controls

Except for alcohol drinking, significant differences in other anthropometric attributes and biochemical profiles were observed between the two groups (Table 1). Compared to the non-DM controls, subjects with DM were significantly older and more obese, had significantly higher blood pressure and Log (FTG), and had significantly lower TCHO, LDL-C, and HDL-C. The DM cases had significantly lower proportions of female sex and schooling years ≥12 years and significantly higher proportions of a positive history of physician-diagnosed hypertension and current cigarette smoking than the non-DM controls.

### 3.2. Comparisons of Carotid RIs and PIs Between DM Cases and Non-DM Controls

Table 2 shows that all RIs and PIs of the extracranial carotid arteries were significantly different between DM cases and non-DM controls. The means of the RIs of the DM cases were 0.017 to 0.020 higher than those of the non-DM controls (all *p*-values < 0.0001). Compared to the non-DM controls, the means of the PIs of the CCA, ICA, and ECA were 0.103, 0.059, and 0.163 higher for the DM cases (all *p*-values < 0.0001).

The differences in the means of the RIs and PIs of the extracranial carotid arteries between the non-DM controls and DM cases decreased but remained statistically significant after controlling for the effects of age and sex (Table 2). Compared to the non-DM controls, the age–sex-adjusted means of the RIs were 0.013 to 0.014 higher for the DM cases (all *p*-values < 0.0001). The age–sex-adjusted means of the PIs of the CCA, ICA, and ECA were 0.065, 0.038, and 0.137 higher for the DM cases compared to those of the non-DM controls (all *p*-values < 0.005).

### 3.3. Association Analyses for Carotid RIs and PIs with the Presence of DM

Table 3 shows that the age–sex-adjusted ORs of having DM were significantly positively correlated with all carotid RIs and PIs. The age–sex-adjusted ORs of having DM ranged from 1.22 to 1.33 and from 1.16 to 1.29 per 1.0 SD increase in the carotid RIs and PIs, respectively (all *p*-values < 0.005).

Multivariable logistic regression analyses of the conventional cardio-metabolic risk factors showed that the most predictive model, i.e., the base model, contained age, sex, hypertension, schooling years, cigarette smoking, BMI, WHR, Log (FTG), TCHO, and HDL-C (Table 4). The multivariable-adjusted ORs of DM were significantly elevated for older age, hypertension, fewer schooling years, cigarette smoking, higher BMI, higher WHR, and higher Log (FTG) and were significantly decreased for higher TCHO and HDL-C. The AUROC of the base model was 0.751 (95% CI, 0.727~0.775).

After controlling for the effects of multiple conventional cardio-metabolic risk factors in the base model, all carotid RIs and PIs remained significantly positively correlated with higher ORs of having DM (Table 3). The associations between DM and the RI and PI of the ECA were stronger than those between DM and the RI and PI of the CCA and ICA. The multivariable-adjusted ORs of having DM per 1.0 SD increase in the ECA RI and PI were 1.36 (95% CI, 1.21~1.54, *p* = 3.9 × 10^−7^) and 1.30 (95% CI, 1.17~1.45, *p* = 8.7 × 10^−7^), respectively. The multivariable-adjusted ORs of having DM remained significant for the RI and PI of the CCA and ICA; but the associations were slightly weaker than those of the age–sex-adjusted estimates.

### 3.4. Analyses for the Added Predictability of Carotid RIs and PIs

Table 3 and Table 4 also show that, compared with the base model, the inclusion of all carotid RIs and PIs improved the predictability of the presence of DM. Compared to the base model, the increases in AUROC ranged from 0.08% to 0.85% and were statistically significant for the inclusion of the ECA RI and PI. The AUROCs for the base + ECA RI and base + ECA PI models were 0.759 (95% CI: 0.736~0.783) and 0.758 (95% CI: 0.7340~0.781), respectively. The corresponding added AUROCs were 0.85% (95% CI, 0.11~1.59%; *p* = 0.025) and 0.69% (95% CI, 0.01~1.37%; *p* = 0.046), respectively.

## 4. Discussion

In this community-based study, we enrolled 402 DM cases and 3416 non-DM controls and explored the relationships between the RI and PI of the extracranial carotid arteries and the likelihood of having DM. We found that, compared to those of the non-DM controls, the means of the carotid RIs and PIs were higher in the DM cases. Additionally, all carotid RIs and PIs were correlated with a significantly higher likelihood of having DM after controlling the effects of conventional CVD risk factors. More importantly, the additions of all carotid RIs and PIs increased the predictability of the presence of DM, and the increments in the AUROC were statistically significant for the ECA RI and PI. To our knowledge, no existing report has assessed the relationships between DM and the segment-specific RIs and PIs of the whole extracranial carotid arteries before.

Previous studies on the relationships between DM and arteries’ RI and PI have frequently measured the CCA and MCA, a few have measured the ICA, and none of them have measured the ECA. In this study, we scanned the extracranial CCA, ICA, and ECA bilaterally and found that all extracranial carotid RIs and PIs are significantly positively correlated with the likelihood of having DM after controlling for the effects of conventional CVD risk factors. Additionally, the RI and PI of the ECA, but not those of the CCA and ICA, are the strongest independent determinants for the presence of DM. Furthermore, the additions of the ECA RI and PI into the base model significantly increase the ability to determine who has and does not have DM. These findings are novel but further validation by other large prospective population-based studies is necessary. The underlying mechanism seems to warrant exploration.

There are many indicators of arterial stiffness, including pulse pressure, PWV, RI, and PI [21,32]. Among these mentioned indicators, PWV is considered the non-invasive ‘gold standard’ measurement of arterial stiffness [12]. However, the relationships between PWV and type II DM are inconsistent. Significant associations between brachial-ankle PWV and DM were observed in the studies by Yiu et al. [16] and Li et al. [17], but not in those by Loimaala et al. [19] and Yoo et al. [20]. A recent meta-analysis study found that aortic PWV is significantly negatively correlated and brachial-ankle PWV is significantly positively correlated with DM [15]. Moreover, the I^2^ statistics are 94.8% and 76.1%, respectively, indicating the existence of significant variation in the effect estimates among studies [15]. In addition, several studies have demonstrated significant associations between PWV and DM; however, none of them have assessed the significance of the increase in discriminatory ability caused by adding PWV to the prediction model [16,17,33,34,35]. Moreover, among the previously mentioned reports, only one study has considered the confounding effects of the conventional CVD risk factors [35]. A meta-analysis study, which included 17,662 subjects and 898 stroke incidences from four prospective studies, showed that higher carotid-femoral PWV, the most frequently used arterial stiffness measurement, is significantly correlated with an elevated risk of incident stroke [14]. However, compared with the base model, the increase in AUROC is non-significant and negligible [14]. This evidence indicates that PWV might not be a major independent determinant of DM-related arterial stiffness, and it seems that it is necessary to search for novel biomarkers.

The PI and RI are indices of vascular stiffness and can be non-invasively measured by Doppler ultrasonography [21]. Recent research has also correlated arterial PI and RI with CVDs. A community-based prospective study found that, compared to the first tertiles of the PI and RI of the CCA, the incidence rates of stroke were significantly elevated for the second and third tertiles [22]. The Oxford Vascular Study showed that the PI of the ICA is significantly correlated with the markers and burden of cerebral small vessel disease [23]. A case–control study showed that, compared to the lowest PI quartile of the basilar artery, the multivariable-adjusted OR of neurological deterioration was 2.39 (95% CI, 1.10~5.25) for the highest quartile [24]. A recent retrospective study included 122 patients with cerebral small vessel occlusion within 24 h of onset and found that high-scoring patients had a significantly higher ICA PI than the low-scoring patients. The difference remained statistically significant after controlling for multiple CVD risk factors [36].

The evidence that the arterial PI and RI are significantly correlated with cerebrovascular diseases indicates that the arterial PI and RI are probably novel markers of DM-related arterial stiffness. Indeed, there are reports that support the roles of the arterial PI and RI in DM-related arterial stiffness. Lee et al. enrolled 56 stroke-free normotensive DM patients and 70 age–sex-matched non-DM controls and found that the means of the PIs of the ICA, MCA, and basilar artery (BA) of the DM patients were significantly higher than those of the non-DM controls [25]. Similarly, Park et al. measured the PI of ICA, MCA, and BA of 30 DM patients with insulin resistance (Group 1), 30 DM patients without insulin resistance (Group 2), and 45 age–sex-matched non-DM controls (Group 3). They found that all PIs of Group 1 were significantly higher than those of Group 2, and all PIs of Group 2 were significantly higher than those of Group 3 [26]. Zou et al. compared the PI and RI of the dorsalis pedis artery and the plantar digital artery of 56 DM patients with those of 50 healthy subjects [27]. Similarly, that study showed that the PIs and RIs of the DM patients were significantly higher than those of the healthy controls [27]. An Australian study also showed that the mean BA PI of DM patients was significantly higher than that of the non-DM controls (0.93 [0.03] vs. 0.79 [0.03]) [28]. Recently, a prospective study of atomic bomb survivors correlated the PI and RI of the CCA with the levels of fasting plasma glucose and insulin, the area under the glucose curve, and the Matsuda index [37]. The correlations between the area under the glucose curve and the CCA PI and RI remained statistically significant after controlling for the effects of conventional CVD risk factors [37]. In summary, previous studies have measured the arterial PI and RI in different territories, and their relationships with DM are consistent and significant. However, most of the mentioned reports are hospital-based and have small sample sizes [25,26,28]. The recent Japanese study is population-based and has a large sample size; however, the study subjects come from a special population [37]. There are no data from population- or community-based studies with large sample sizes from the general population.

This study has several strengths. First, the significant relationships between the RI and PI of the ECA and DM are novel findings. All carotid RIs and PIs increase the ability to determine who will and who will not have DM. More importantly, compared to the base model, the increments in the discriminatory ability are statistically significant for the ECA RI and PI. On the contrary, while the RI and PI of the CCA and ICA are significantly independent determinants of DM, they do not significantly increase the discriminatory ability. Secondly, compared to previous studies on DM with arterial PI and RI, this study has the largest sample size and recruited subjects from the general population. In this study, all subjects had never received a carotid ultrasound scan before and had no prior CVD history. Therefore, our findings are more likely to reflect the natural spectrum. Thirdly, we assessed the independent effects of extracranial carotid RIs and PIs by controlling for 10 conventional CVD risk factors. The relationships between DM and carotid RIs and PIs are probably valid. Lastly, in this study, the directions of the effects of carotid RIs and PIs with the likelihood of having DM are the same. Our findings are internally consistent.

This study has three potential limitations. The first is that the present study is observational in nature, which indicates that causal inference must be made with caution. The second is that we found that the ECA RI and PI are more significant and predictive than those of the CCA and ICA. However, due to the structure of the ECA, it is difficult to find a suitable site to measure the blood flow velocity. As a result, data regarding the ECA RI and PI are more likely to be missed than data regarding the RI and PI of the CCA and ICA. Among the 3908 subjects who received carotid duplex scans in this study, the blood flow velocities of the CCA and ICA were unavailable for 16 (0.41%) and 42 (1.07%) subjects, respectively. However, as many as 82 (2.10%) subjects lacked data related to the ECA. The third limitation is that DM is a complex disease and correlates with multiple complications [4,5,6,7]. Many modifiable and un-modifiable factors have been correlated with DM [8,9]. This study could only explore the effects of a limited number of them. The last limitation is that due to the implementation of the Personal Information Protection Act, the district offices of household registration are prohibited from providing us with detailed information regarding the households with eligible subject(s). The only information available is the address of each household with eligible subject(s). Consequently, we were unable to evaluate the representativeness and response rate of the study sample. However, the primary aim of the study was to explore the relationship between DM and carotid RIs and PIs. Furthermore, all subjects were unaware of the study’s hypotheses and did not receive a carotid ultrasound scan before enrollment. Accordingly, our findings should be conservative.

## 5. Conclusions

We found that all RIs and PIs of the extracranial carotid arteries are significantly correlated with the likelihood of having DM. All carotid RIs and PIs also increase the ability to determine who will and will not have DM. More importantly, the increased predictability of the ECA RI and PI was statistically significant. The RI and PI of the ECA are probably novel biomarkers of DM-related vascular diseases.

## Figures and Tables

**Table 1:** Comparisons of anthropometric characteristics and biochemical profiles between DM patients and non-DM controls.

Variables	Non-DM Controls (n = 3416)		DM Patients (n = 402)		*p*-Values
Continuous variables	Mean	SD	Mean	SD	
Age (years)	55.5	8.9	60.0	8.4	<0.0001
BMI (kg/m^2^)	24.3	3.4	26.1	3.8	<0.0001
Waist circumference (cm)	84.8	10.0	90.3	9.8	<0.0001
Hip circumference (cm)	96.2	7.1	98.0	7.7	<0.0001
WHR (%)	88.1	7.1	92.1	6.6	<0.0001
SBP (mmHg)	125.7	18.6	131.4	17.1	<0.0001
DBP (mmHg)	76.0	12.6	78.2	12.1	0.0012
TCHO (mg/dL)	206.0	37.6	196.9	43.7	<0.0001
LDL (mg/dL)	122.2	32.1	115.6	34.7	0.0003
HDL (mg/dL)	56.5	15.0	49.1	12.6	<0.0001
LDL-/HDL-C ratio	2.31	0.84	2.48	0.89	0.0001
Log (FTG)	4.57	0.55	4.86	0.58	<0.0001
Categorical variables	n	%	n	%	
Female sex	2259	66.0	222	55.2	<0.0001
Schooling years ≥ 12 years	1308	38.3	111	27.6	<0.0001
Hypertension	715	20.9	175	43.5	<0.0001
Current cigarette smoker	709	20.8	120	29.9	<0.0001
Current alcohol drinker	472	13.9	57	13.2	0.86

Note: BMI, body mass index; DBP, diastolic blood pressure; DM, diabetes mellitus; FTG, fasting plasma triglycerides; HDL-C, high-density lipoprotein cholesterol; LDL-C, low-density lipoprotein cholesterol; SBP, systolic blood pressure; SD, standard deviation; TCHO, total cholesterol; WHR, waist-to-hip ratio.

**Table 2:** Effects of DM on hemodynamic parameters of extracranial carotid arteries.

	Non-DM Controls (n = 3416)		DM Patients (n = 402)		Difference Between DM Cases and Non-DM Controls			
					Crude		Age–Sex-Adjusted	
Indicator	Mean	SD	Mean	SD	Mean	(95% CI)	Mean	(95% CI)
CCA								
RI	0.72	0.05	0.74	0.05	0.020 ***	(0.015~0.026)	0.013 ***	(0.008~0.018)
PI	1.52	0.32	1.62	0.34	0.103 ***	(0.070~0.136)	0.065 ***	(0.034~0.096)
ICA								
RI	0.60	0.06	0.62	0.06	0.020 ***	(0.013~0.026)	0.013 ***	(0.006~0.019)
PI	1.03	0.23	1.09	0.23	0.059 ***	(0.035~0.083)	0.038 **	(0.014~0.062)
ECA								
RI	0.80	0.06	0.82	0.06	0.017 ***	(0.011~0.024)	0.014 ***	(0.007~0.020)
PI	1.99	0.48	2.15	0.54	0.163 ***	(0.113~0.214)	0.137 ***	(0.087~0.186)

Note: CCA, common carotid artery; CI, confidence interval; DM, diabetes mellitus; ECA, external carotid artery; ICA, internal carotid artery; OR, odds ratio; PI, pulsatility index; RI, resistance index; SD, standard deviation. **, 0.0001 < *p* < 0.005; ***, *p* < 0.0001.

**Table 3:** Associations of DM with resistance index and pulsatility index of extracranial carotid arteries.

Indicators (per SD)	Age–Sex-Adjusted		Multivariable-Adjusted ^1^		Added AUROC (%) ^2^	
	OR	(95% CI)	OR	(95% CI)	Δ AUROC	(95% CI)
CCA						
RI	1.33 ***	(1.18~1.50)	1.25 **	(1.11~1.42)	0.26	(−0.32~0.83)
PI	1.23 ***	(1.11~1.37)	1.15 *	(1.02~1.29)	0.10	(−0.24~0.45)
ICA						
RI	1.22 **	(1.10~1.36)	1.22 **	(1.09~1.37)	0.24	(−0.27~0.76)
PI	1.16 **	(1.05~1.28)	1.14 *	(1.02~1.26)	0.08	(−0.23~0.38)
ECA						
RI	1.25 ***	(1.11~1.40)	1.36 ***	(1.21~1.54)	0.85 *	(0.11~1.59)
PI	1.29 ***	(1.16~1.42)	1.30 ***	(1.17~1.45)	0.69 *	(0.01~1.37)

^1^ Adjusted for age, sex, education, cigarette smoking, hypertension, BMI, WHR, Log (FTG), TCHO, and HDL-C. ^2^ Compared with model containing age, sex, education, cigarette smoking, hypertension, BMI, WHR, Log (FTG), TCHO, and HDL-C. Note: AUROC, area under the receiver operating characteristic curve; CCA, common carotid artery; CI, confidence interval; DM, diabetes mellitus; ECA, external carotid artery; ICA, internal carotid artery; OR, odds ratio; PI, pulsatility index; RI, resistance index; SD, standard deviation. *, 0.005 < *p* < 0.05; **, 0.0001 < *p* < 0.005; ***, *p* < 0.0001.

**Table 4:** Multivariable analyses for DM with conventional cardio-metabolic risk factors.

	Base Model		Base + ECA RI		Base + ECA PI	
Variable	OR	(95% CI)	OR	(95% CI)	OR	(95% CI)
Age (per 10 years)	1.50 ***	(1.32~1.70)	1.41 ***	(1.24~1.60)	1.45 ***	(1.28~1.64)
Sex (M/F)	0.89	(0.67~1.17)	0.78	(0.59~1.03)	0.75 *	(0.57~1.00)
Schooling years < 12 years (Y/N)	1.39 *	(1.09~1.78)	1.38 *	(1.08~1.76)	1.40 *	(1.10~1.80)
Cigarette smoking (Y/N)	1.35 *	(1.01~1.81)	1.42 *	(1.06~1.90)	1.39 *	(1.04~1.87)
Hypertension (Y/N)	1.50 **	(1.18~1.90)	1.46 **	(1.15~1.85)	1.44 **	(1.14~1.84)
BMI (per 1.0 SD)	1.25 **	(1.11~1.40)	1.24 **	(1.10~1.39)	1.22 **	(1.08~1.37)
WHR (per 1.0 SD)	1.27 **	(1.12~1.45)	1.33 ***	(1.17~1.52)	1.30 ***	(1.14~1.49)
TCHO (per 1.0 SD)	0.79 **	(0.70~0.90)	0.78 **	(0.69~0.89)	0.79 **	(0.69~0.90)
HDL-C (per 1.0 SD)	0.83 *	(0.70~0.98)	0.84 *	(0.71~0.99)	0.83 *	(0.70~0.98)
Log (FTG) (per 1.0)	1.88 ***	(1.47~2.40)	1.95 ***	(1.53~2.50)	1.92 ***	(1.51~2.46)
ECA RI (per 1.0 SD)	-		1.36 ***	(1.21~1.54)	-	
ECA PI (per 1.0 SD)	-		-		1.30 ***	(1.17~1.45)
AUROC (%)	75.1	(72.7~77.5)	75.9	(73.6~78.3)	75.8	(73.4~78.1)

Note: AUROC, area under the receiver operating characteristic curve; BMI, body mass index; CI, confidence interval; DM, diabetes mellitus; ECA, external carotid artery; FTG, fasting plasma triglycerides; HDL-C, high-density lipoprotein cholesterol; PI, pulsatility index; OR, odds ratio; RI, resistance index; SD, standard deviation; TCHO, total cholesterol; WHR, waist-to-hip ratio. *, 0.005 < *p* < 0.05; **, 0.0001 < *p* < 0.005; ***, *p* < 0.0001. -, not included.

## Data Availability

The datasets used and/or analyzed during the current study are available from the corresponding author upon reasonable request.

## Abbreviations

The following abbreviations are used in this manuscript:

DM	diabetes mellitus
PI	pulsatility index
RI	resistance index
CCA	common carotid arteries
ICA	internal carotid arteries
ECA	external carotid arteries
OR	odds ratio
CI	confidence interval
SD	standard deviation
DALY	disability-adjusted life year
PWV	pulse wave velocity
SBP	systolic blood pressure
DBP	diastolic blood pressure
HDL-C	high-density lipoprotein cholesterol
LDL-C	low-density lipoprotein cholesterol
TCHO	total cholesterol
FTG	fasting triglyceride
FPG	fasting plasma glucose
PSV	peak-systolic velocity
EDV	end-diastolic velocity
MFV	time-weighted maximum flow velocity
AUROC	area under the receiver operating characteristic curve
